# Utilizing the Potential of Waste Hemp Reinforcement: Investigating Mechanical and Thermal Properties of Polypropylene and Polylactic Acid Biocomposites

**DOI:** 10.1021/acsomega.3c06240

**Published:** 2024-02-14

**Authors:** Anıl Yılmaz, Hakan Özkan, F. Elif Genceli Güner

**Affiliations:** † Department of Chemical Engineering, Istanbul Technical University, 34469 Maslak, Istanbul, Turkey; ‡ Arçelik Çayırova Campus, R&D Material Technologies, R&D Center, 34950 Istanbul, Turkey; § Polar Research Center (PolReC), Istanbul Technical University, 34469 Maslak, Istanbul, Turkey

## Abstract

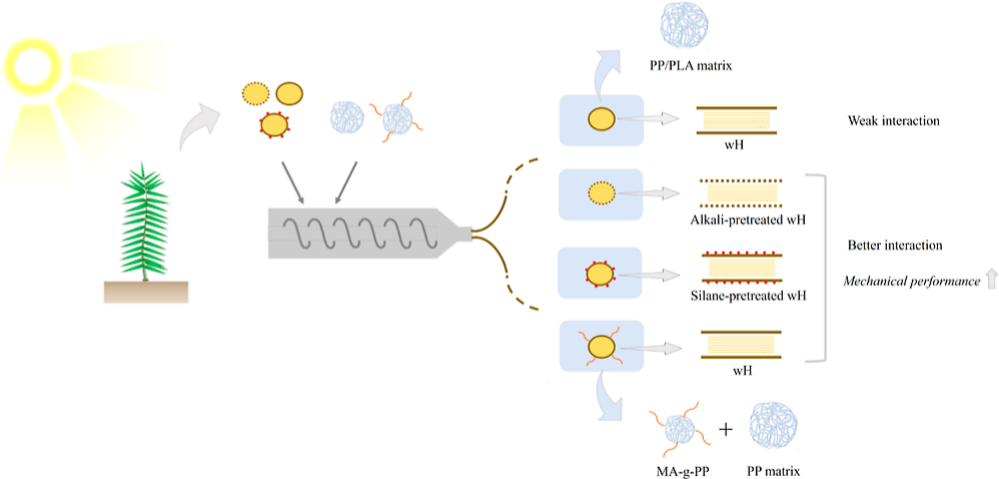

Hemp has gained significant popularity for its diverse applications; however, this study explores the untapped potential of waste hemp (wH) as a cost-effective and sustainable bioadditive for the development of high-performance biocomposites. wH offers advantages such as low cost, easy availability, and suitability for extrusion. Polypropylene (PP) and poly(lactic acid) (PLA) served as polymer matrices for this investigation. In order to enhance the interaction between the wH and polymer matrices, alkaline and silane pretreatments were applied to the wHs of both matrices. At the same time, the MA-*g*-PP additive was used exclusively for the PP matrix. The resulting PP biocomposite demonstrated Young’s modulus (2986 MPa) and flexural modulus (2490 MPa), surpassing those of neat PP by 109 and 77%, respectively. Similarly, wH40-PLA-A showed enhancements in the PLA biocomposite, with Young’s modulus (6214 MPa) and flexural modulus (5970 MPa) representing an increase of 81 and 56% over that of neat PLA, respectively. The thermal properties and behaviors of the resulting biocomposites were minimally affected by the inclusion of wH as a bioadditive. This study contributes to the advancement of sustainable materials and provides valuable insights into the utilization of wH as a valuable resource for the development of high-performance biocomposites.

## 1 Introduction

In recent years, utilization of biomass as a valuable resource for various applications has gained significant attention.^1−5^ One promising avenue is its incorporation into polymer composites, where biomass can serve as a reinforcement such as fibers^6−11^ or fillers.^12−15^ Despite its ecological and economic advantages, biomass has disadvantages such as variability in its properties, depending on climate and growing conditions. Among the key drawbacks encountered in polymer composite applications, the issue of incompatibility at the interface between biomass and the polymer matrix is prominent. This incompatibility arises from the inherently hydrophilic nature of biomass and the hydrophobic nature of polymer matrices, which can lead to the development of composite materials characterized by poor mechanical properties.^16−19^ Thus, it is necessary to make biomass compatible with the polymer matrix by using various pretreatments and additives. Biomass-matrix interfacial adhesion can be improved by modifying either biomass or the polymer.^20^ Modification of the polymer is also achieved by adding a coupling agent to improve adhesion to the matrix and biomass. MA-*g*-PP is one of the most suitable coupling agents for use in biomass-based fiber (BBF)-reinforced polypropylene composites.^21−23^ MA-*g*-PP is chemically bonded to lignocellulosic materials through MA groups, and the polymer chains in its structure are bonded to the matrix. Thus, it acts as a bridge between the nonpolar polymer and the polar BBF.^20^ For this reason, MA-*g*-PP is used as a coupling agent to increase the compatibility between the biomass and polymer.^24,25^ Another method for enhancing the biomass-matrix interface is alkaline pretreatment. It is the oldest known method for cellulose-based modification of natural substances.^26^ With this pretreatment, hemicellulose, lignin, and various impurities are removed from the lignocellulosic structure.^27,28^ As a result, the interfibrillar region becomes less dense and less rigid, allowing some rearrangement of the cellulosic fibrils.^29^ Hemicellulose is the most hydrophilic part of the biomass structure; therefore, the biomass surface becomes more hydrophobic. In silane pretreatment, a silane coupling agent is added to the biomass surface, which forms the biomass-matrix connection. These agents perform two functions. The first is to react with the –OH groups of lignocellulosic biomasses and the second is to react with the functional groups of the polymer matrix.^30,31^ Sullins et al.^6^ studied the effects of material treatment(s) on the mechanical behaviors of hemp fiber-reinforced polypropylene (PP) composites. The hemp fiber was supplied in the mat form. Hemp fiber-reinforced PP composites were prepared using compounding-extrusion-compression molding processes. Mechanical behaviors were investigated using various material treatment(s) combinations, including 5% maleic anhydride (MA)-grafted polypropylene (MA-*g*-PP), 5% NaOH-treated hemp fiber, 10% NaOH-treated hemp fiber, and 5% NaOH + 5% MA-*g*-PP. In composites with these material treatments, hemp fiber contents of 15 and 30% were used. Both composites with MA-*g*-PP only, 15–5 MA-*g*-PP, and 30–5 MA-*g*-PP, outperformed the other material variations with the same fiber content. Sawpan et al.^7^ examined the flexural strength and flexural modulus of chemically treated random short and aligned long hemp fiber-reinforced polylactide and unsaturated polyester composites at various fiber contents. Industrial hemp fibers were used in this study. PLA/short fiber composites were compounded (10, 20, and 30 wt % fiber) in a twin-screw extruder and then injection molded. The alkali and silane treatments on the fibers were found to enhance the flexural strength and flexural modulus of the composites, potentially attributed to improved adhesion between the fibers and the matrix. Mazzanti et al.^8^ examined the role of hemp fiber morphology in the PLA/hemp system. In the study, it was determined that the mechanical properties increased as a result of alkali pretreatment. It also demonstrates that favorable mechanical results can be achieved in the hemp-PLA system, providing the effective dispersion of hemp bundles into individual fibers. It is emphasized that for good mechanical properties, in addition to improving the fiber–matrix interface, the number of fiber bundles in the matrix should also be minimized. Nanni et al.^15^ investigated the functionalization and use of grape stalks as poly(butylene succinate) (PBS) reinforcing fillers. Grape stalks were collected from wine cellars in northern Italy. Biocomposites were prepared by extrusion using 10 wt % grape stalks. The biocomposites exhibited higher stiffness than the control polymer, as evidenced by an increase in Young’s modulus from 616 to 732 MPa in the specimens fabricated using acetylated grape stalk powder. Gupta et al.^12^ used hemp powder (HP), a byproduct of the bast hemp fiber production process and they investigated it as a functional additive of polybutylene adipate-*co*-terephthalate (PBAT) resins to produce biocomposites. HP-filled PBAT biocomposites were developed through extrusion and injection molding. The addition of MA-modified PBAT and its integration into the biocomposite resulted in a substantial enhancement in the tensile strength (209%) of the biocomposite containing 40% HP.

Most hemp used in the literature was of industrial grade or cultivated for particular purposes. On the other hand, in our study, waste hemp (wH) was used as a polymer additive to convert it into a value-added product. Our motivation is to evaluate the wH, derived from pelletizing all of the smallest particles left after processing the fibrous stalks of the *Cannabis sativa* plant. The useless lignocellulosic residues left after processing are economical and are easily accessible. Therefore, the reuse of this abundant waste material and its integration into the polymer structure are remarkable, as they are obtained from renewable resources in a sustainable manner. The incorporation of wH into polymer composites represents a mutually beneficial solution; it offers an effective means of managing wH while concurrently enhancing the performance and minimizing the environmental footprint of polymers. The mechanical properties of polymer matrices can be greatly improved by adding wH as a reinforcement. The utilization of wH in our composite material significantly enhances the economic aspects of the process. wH, which is often an underutilized byproduct, is a valuable resource. By repurposing this abundant waste material, we reduced the need for costly raw materials, such as petroleum-based fillers. This not only lowers production costs but also contributes to a more sustainable and cost-effective manufacturing process. This study investigates the impact of varying wH content (10, 20, 30, and 40 wt %) and different pretreatment methods (alkaline, silane, and MA-*g*-PP) on the mechanical, thermal, and interfacial characteristics of PP and PLA biocomposites, utilizing wH as a reinforcement element.

## 2 Materials and Methods

Borealis HE125MO PP (Borealis AG, Austria) was used as a polymer matrix. PLA L175, used as a polymer matrix, was supplied by Total SA (Corbion, Holland). This study used wH as a reinforcing element added to polymer matrices. The wH was supplied by Natural Fiber (Lithuania). Proximate analysis results of the wH are summarized in Table S1. Using an optical microscope, the average length and diameter of the fibers in the wHs were determined to be 2.1 ± 0.1 mm and 68.0 ± 8.8 μm, respectively. Figure S1 displays the image obtained from the optical microscope. The MA-*g*-PP coupling agent (used at 2 wt %) was provided by BYK, Germany. MA-*g*-PP is a coupling agent with MA grafted into a 1.8 wt % chemically bonded PP matrix. The formation of acidic or basic groups as a result of hydrolysis in PLA accelerates hydrolysis via an autocatalytic effect. Therefore, a stabilizer was added to the PLA structure to provide a hydrolytic resistance. The formulation of this stabilizer is particular and produced by Arçelik A.Ş. Triethoxy (3,3,4,4,5,5,6,6,7,7,8,8,8-tridecafluorooctyl) silane, a silane coupling agent, from Evonik Industries AG, Germany, was used to increase the compatibility of the hydrophobic polymer matrix and the hydrophilic wH. Sodium chlorite was supplied by Sigma-Aldrich, USA. Benzene, ethanol, acetic acid, and sulfuric acid were provided by Merck, Germany.

### 2.1 Pretreatment of the Waste Hemp

**Figure 1:**
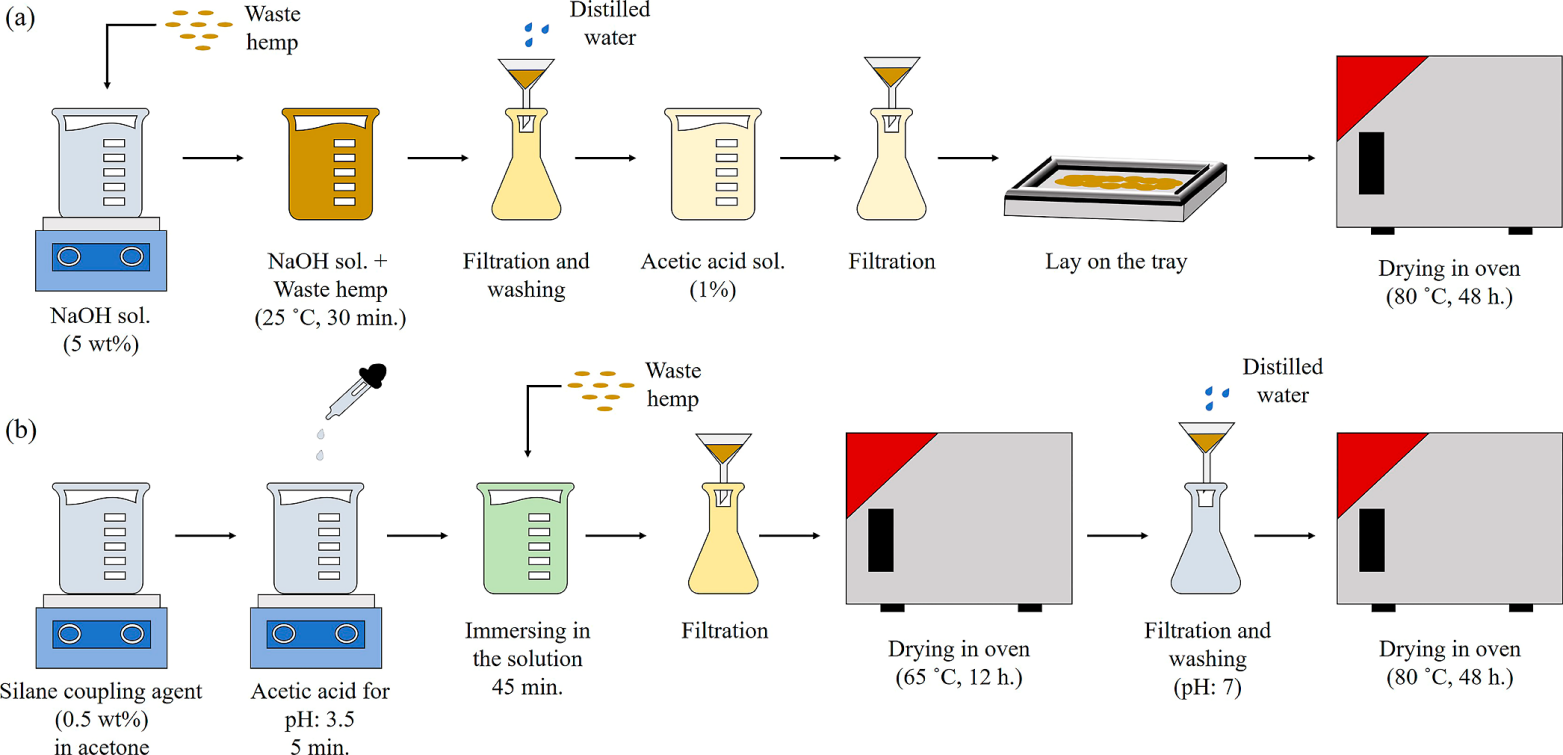
Schematic representation of (a) alkaline and (b) silane pretreatment.

Before preparing the biocomposites, alkaline or silane pretreatments were applied to the wHs using the procedure of Sawpan et al.^37^ For alkaline pretreatment, the wHs were soaked in a 5 wt % NaOH solution at room temperature for 30 min. Then, the wHs were washed with water to eliminate all alkali residues on the fiber surfaces and subsequently neutralized with a 1 wt % acetic acid solution. After that, the pretreated wHs were dried in an oven at 80 °C for 48 h (Figure 1a). For silane pretreatment, a solution of 0.5 wt % silane coupling agent was prepared in acetone, and the pH of the solution was adjusted to 3.5 with acetic acid. The wHs were then immersed in this solution for 45 min. The wHs were then removed from the solution and dried in an oven at 65 °C for 12 h. Finally, the wHs were washed with distilled water to eliminate chemical impurities until a pH of 7 was reached and then dried in an oven at 80 °C for 48 h (Figure 1b).

### 2.2 Biocomposite Preparation

**Figure 2:**
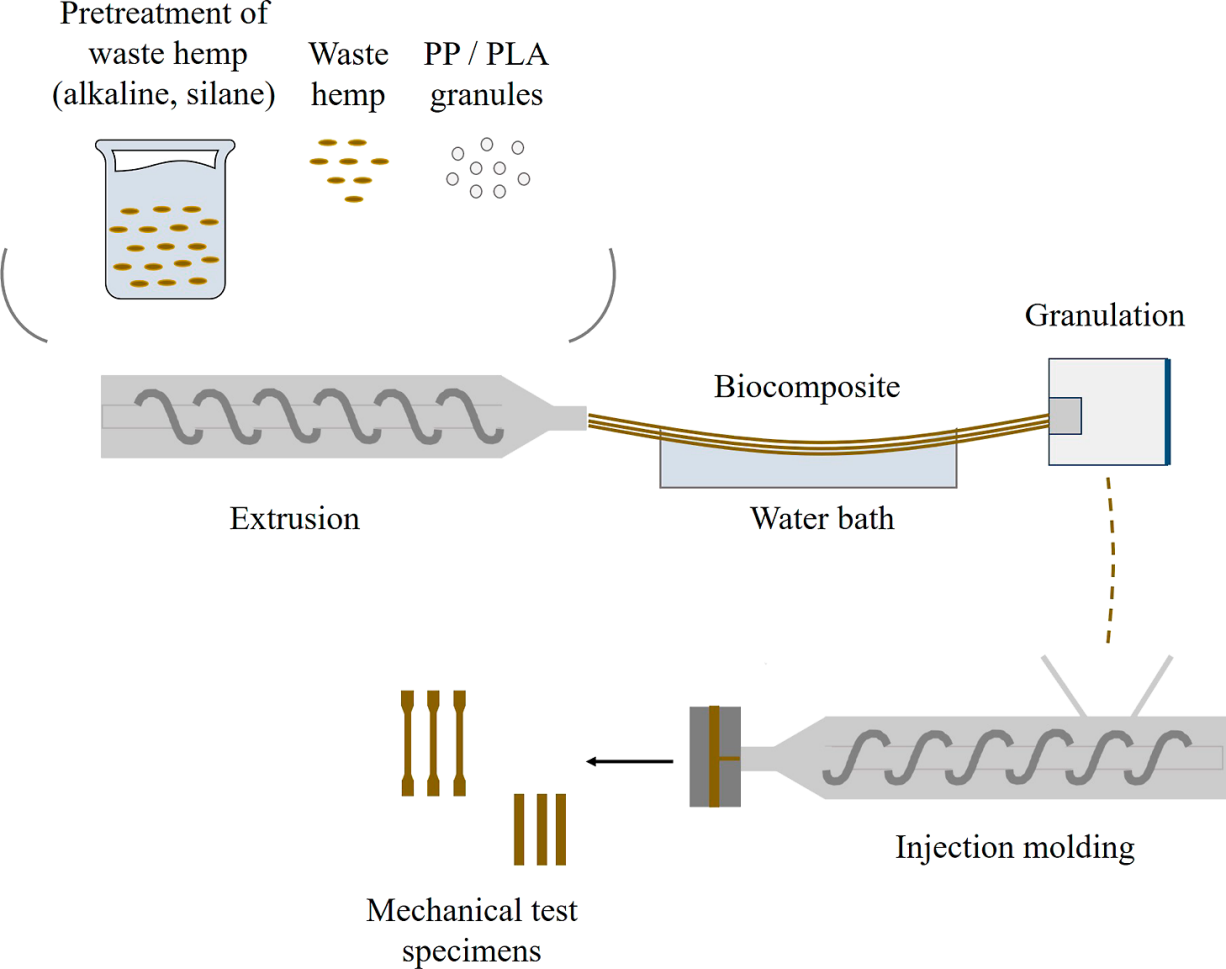
Process from pretreated wHs to obtain mechanical test specimens.

Extrusion and injection molding procedures for pretreated and nonpretreated wHs were performed to characterize the samples and perform mechanical tests (Figure 2).

The wH-reinforced biocomposites were prepared by using a twin-screw PRISM TSE24 IIC extruder at a screw speed of 200 rpm with a screw diameter of 24 mm and an L/D ratio of 28:1 (shaft length/screw diameter). Since wH was used, it was set at 170 °C/175 °C/180 °C/185 °C/190 °C/195 °C/200 °C extruder temperature profiles throughout the feed-exit zone. Formulations including alkaline (A) and silane (S) pretreatment that were produced as a granular form in the compound extruder are listed in Tables 1 and 2. An ARBURG brand 320 C 500–250 injection device was used. The biocomposite materials formed in granular form were then molded by using an injection device and standardized test specimen molds to perform mechanical tests. On the other hand, MA-*g*-PP was added directly to the PP matrix at 2 wt % in the extrusion process. The wHs were then added to the extruder without any pretreatment.

**Table 1:** Sample Codes and Compositions of the wH/PP Biocomposites

sample code	wH (wt %)	A	S	MA-*g*-PP
wH10-PP	10	–	–	–
wH10-PP-MA	10	–	–	+
wH10-PP-S	10	–	+	–
wH10-PP-A	10	+	–	–
wH20-PP	20	–	–	–
wH20-PP-MA	20	–	–	+
wH20-PP-S	20	–	+	–
wH20-PP-A	20	+	–	–
wH30-PP	30	–	–	–
wH30-PP-MA	30	–	–	+
wH30-PP-S	30	–	+	–
wH30-PP-A	30	+	–	–
wH40-PP	40	–	–	–
wH40-PP-MA	40	–	–	+
wH40-PP-S	40	–	+	–
wH40-PP-A	40	+	–	–

**Table 2:** Sample Codes and Compositions of wH/PLA Biocomposites

sample code	wH (wt %)	A	S
wH10-PLA	10	–	–
wH10-PLA-S	10	–	+
wH10-PLA-A	10	+	–
wH20-PLA	20	–	–
wH20-PLA-S	20	–	+
wH20-PLA-A	20	+	–
wH30-PLA	30	–	–
wH30-PLA-S	30	–	+
wH30-PLA-A	30	+	–
wH40-PLA	40	–	–
wH40-PLA-S	40	–	+
wH40-PLA-A	40	+	–

### 2.3 Characterization

Scanning electron microscopy (SEM) analyses were performed by a Zeiss SEM instrument at an acceleration voltage of 5 kV. The specimens were fractured under liquid nitrogen and gold-sputtered prior to observation. Fourier transform infrared (FTIR) analyses were carried out using an Excalibur series FTS 3000 MX FTIR spectrometer with a diamond crystal ATR adapter in the wavelength range of 4000–600 cm^–1^. Differential scanning calorimetry (DSC) measurements were executed using a TA Instruments DSC Q-2000. About 10 mg of the compound sample was sealed in aluminum pans, and an empty aluminum pan and lid were used as references. Two heating and cooling scans were carried out at 10 °C/min in flowing nitrogen at a flow rate of 50 mL/min. Thermogravimetric analysis (TGA) was conducted using a PerkinElmer Diamond TG/DTA thermogravimetric analyzer under a nitrogen flow (100 mL/min) at a heating rate of 10 °C/min. Opticam microscope measurements were performed using a Zeiss Axio Imager at 5× magnification.

## 3 Mechanical Tests

Tensile tests were performed at room temperature in accordance with ISO 527 standards using a Zwick Z020 universal tensile testing machine equipped with a 20 kN load cell. The cross-head speed was maintained at 50 mm/min. Bending tests were carried out in three-point bending mode. An Instron 4505 universal testing machine equipped with a 5 kN load cell was used, and the tests were conducted in accordance with ISO 78 standards at room temperature. The tests were performed at a cross-head speed of 5 mm/min.

## 4 Results and Discussion

### 4.1 Determining the Presence of Pretreatments

**Figure 3:**
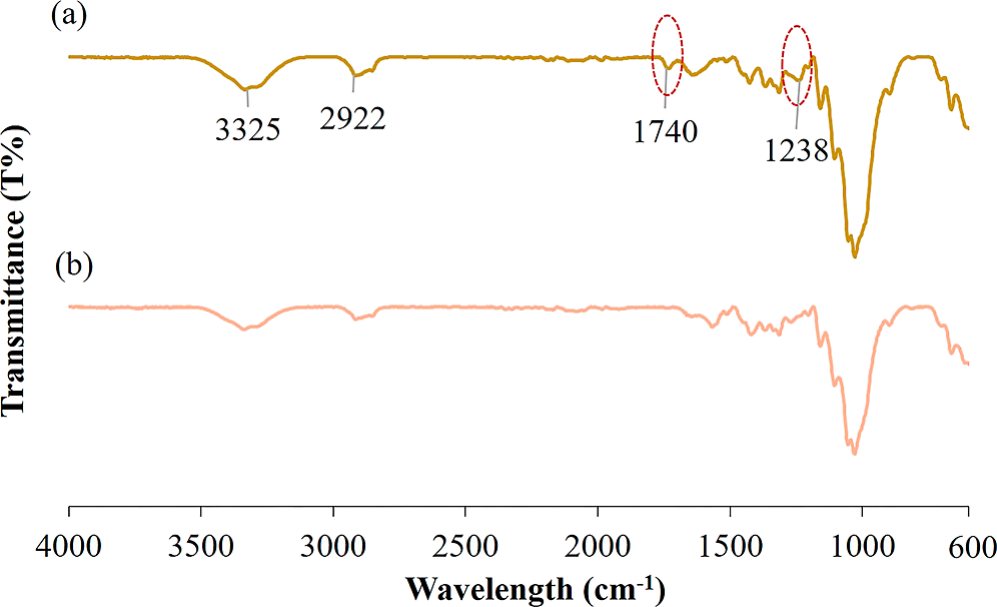
FTIR spectrum of (a) nonpretreated and (b) alkaline-pretreated wHs.

The FTIR spectra of the waste wHs with and without pretreatment are shown in Figure 3. The main components of lignocellulosic biomass are cellulose, lignin, and hemicelluloses. FTIR spectral bands obtained depend mainly on these components. The peak at 3325 cm^–1^ in untreated wHs is due to the O–H stretching vibration in hemicellulose or cellulose as well as absorbed moisture. The peak at 2922 cm^–1^ corresponds to the C–H stretching vibration of the –CH_2_ group in the cellulose and hemicellulose structure. The peak at 1740 cm^–1^ indicates the C–O stretching vibration of the carbonyl groups in hemicellulose, while the peak at 1238 cm^–1^ is a C–O stretching of the acetyl groups of lignin.^38,39^

The most important changes in the FTIR spectrum of alkaline-pretreated wHs were the absence of characteristic peaks at 1740 and 1238 cm^–1^ associated with hemicellulose and lignin. For alkaline-pretreated wH, the peak at 1740 cm^–1^ disappeared, indicating that the ester group in hemicellulose was easily removed by the alkaline pretreatment. As a result, it can be summarized that the pretreatment with alkali removes most of the hemicellulose and lignin components. In addition, the process hydrophobically changes the hydrophilic structure of wH. FTIR spectra do not clearly demonstrate the effect of silane pretreatment on transmittance bands. This can be attributed to the relatively low concentration of silane present on the wH surfaces, which fell below the detectable threshold of the FTIR analysis.^39,40^

**Figure 4:**
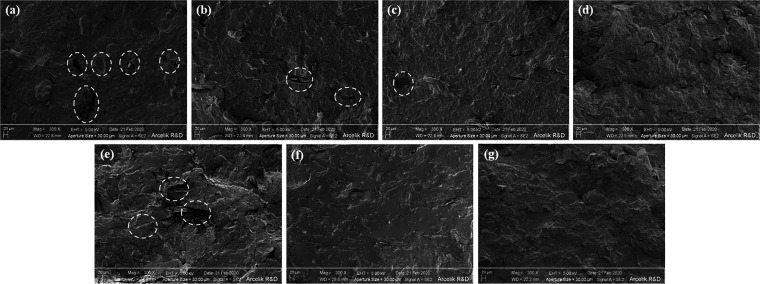
SEM images of (a) nonpretreated (wH20-PP), (b) alkaline-pretreated (wH20-PP-A), (c) MA-*g*-PP additive (wH20-PP-MA), and (d) silane-pretreated (wH20-PP-S); (e) nonpretreated (wH20-PLA), (f) alkaline-pretreated (wH20-PLA-A), and (g) silane-pretreated (wH20-PLA-S) samples.

The SEM images of wH20-PP, wH20-PP-A, wH20-PP-MA, and wH20-PP-S are shown in Figure 4a–d. The characteristic wH-matrix interphase was clearly observed for the 20% wH-content biocomposite. Interphase decomposition was observed at the wH-matrix interface of wH20-PP. Most of the large voids in the matrix are formed by the removal of incompatible wH from the structure during cracking for analysis. Some holes on the wH surface also indicate incompatibility with the polymer. It is seen that there are more gaps in wH20-PP than in the others owing to decomposition of the wH matrix. Figure 4a–d shows that all pretreated PP samples had fewer voids and were better trapped than the untreated composite. Figure 4e–g displays SEM images of wH20-PLA, wH20-PLA-A, and wH20-PLA-S. Decomposition was observed at the wH-matrix interface of wH20-PLA. The wH20-PLA-A and wH20-PLA-S biocomposites had greater compatibility and wettability than the pretreated PP biocomposites.

**Figure 5:**
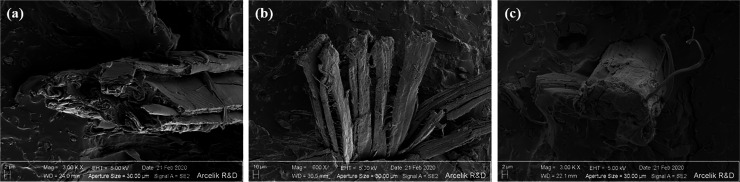
SEM images of (a) nonpretreated, (b) alkaline-pretreated, and (c) silane-pretreated wH.

Figure 5 indicates nonpretreated wH, alkaline-pretreated wH, and silane-pretreated wH. Removing most of the binding materials, such as hemicellulose and lignin, from alkaline-pretreated wHs (Figure 5b) made wH rougher. In fact, most fibrils in the pretreated wH structure were separated from the bundles standing together.^39^ In silane-pretreated wHs (Figure 5c), silane coupling agent molecules effectively accumulated on the wH surface.

In order to better understand the change in macromolecular ingredients as a result of alkaline pretreatment, the macromolecular compositions of wH and alkaline-pretreated wH were determined by analytical methods. To eliminate extractive components from biomass and acquire an extractive-free sample, the ASTM D1105 standard was employed using a benzene-ethanol extraction method. The process involved subjecting wH (according to the results of the sieve analysis, ∼90% of the particles are in the size range of 0.250–2 mm) to leaching by benzene and ethanol in a Soxhlet extractor. Subsequently, benzene was separated, and the remaining substance was filtered and washed with ethanol and hot water until all traces of the solvents were eliminated. The content of the extractives was determined based on the mass loss of the initial biomass. Extractives-free biomass was used as the starting material to separate the holocellulose and lignin. To isolate holocellulose, which comprises hemicelluloses and celluloses, mixtures of NaClO_2_, acetic acid, and deionized water were completed.^41^ Since there are different cellulosic structures in biomass, it is not possible to determine the percentage of cellulose exactly. Therefore, the holocellulose content was determined using the relevant method. On the other hand, the van Soest method was employed to isolate lignin,^42^ wherein the extractives-free sample was treated with 72 vol % sulfuric acid to hydrolyze the holocellulose and isolate the lignin.^43^ The quantity of acid-insoluble lignin was determined by drying and ashing the neutralized wH.^44^Table 3 indicates the main molecular analysis results for wH and alkaline-pretreated wH.

**Table 3:** Main Molecular Components of wH and Alkali-Treated wH

sample	extractives (%)	holocellulose (%)	lignin (%)	ash (%)
wH	20.4	57.1	15.7	6.8
alkali-treated wH	31.8	62.4	1.1	4.8

The removal of lignin from the structure by alkaline pretreatment was supported by these analyses. During the alkali pretreatment, the decomposed lignin was separated from the structure as an extract, and it was observed that the percentage of extractive matters increased.^45^ FTIR and SEM analyses have proven that hemicellulose is removed from the structure. Therefore, hemicellulose as the percentage of holocellulose is considered significantly low.

### 4.2 Thermal Properties

The melting temperature (*T*_m_), melting enthalpy (Δ*H*_m_), crystallization temperature (*T*_c_), crystallization enthalpy (Δ*H*_c_), and percent crystallinity (χ_c_) of the wH/PP biocomposites are listed in Table 4.

**Table 4:** Thermal Properties of wH/PP Biocomposites

sample code	*T*_m_ (°C)	Δ*H*_m_ (J/g)	*T*_c_ (°C)	Δ*H*_c_ (J/g)	χ_c_
neat PP	164.3	97.8	117.2	99.5	46.8
wH10-PP	165.7	82.9	116.9	97.5	44.1
wH10-PP-MA	166.1	104.4	121.7	110.2	55.5
wH10-PP-A	165.9	95.9	117.8	99.9	51.0
wH10-PP-S	166.4	106.3	118.7	105.5	56.5
wH20-PP	165.5	62.7	117.8	70.2	37.5
wH20-PP-MA	169.4	63.4	121.3	77.0	37.9
wH20-PP-A	165.5	46.9	117.9	55.0	28.1
wH20-PP-S	165.7	63.4	117.8	77.0	37.9
wH30-PP	167.1	60.5	118.4	68.6	41.4
wH30-PP-MA	166.7	69.6	124.9	79.8	47.6
wH30-PP-A	165.5	42.3	117.7	55.4	28.9
wH30-PP-S	167.1	44.3	118.2	50.4	30.3
wH40-PP	164.1	54.1	122.3	53.5	43.1
wH40-PP-MA	168.1	42.9	124.4	46.8	34.2
wH40-PP-A	168.3	46.3	118.5	52.8	36.9
wH40-PP-S	167.3	46.4	118.1	53.6	37.0

The crystallinity (χ_c_) of PP can be obtained using eq 1 as follows^46^
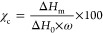
1where Δ*H*_m_ is the measured enthalpy of fusion, Δ*H*_0_ is the enthalpy of fusion for 100% crystalline PP (Δ*H*_0_ = 209 J/g), and ω is the weight fraction of PP in the biocomposites. The calculated χ_c_ values are listed in Table 4. The pretreatments had no significant effect on the thermal properties of the PP biocomposites. Only the addition of MA-*g*-PP increased the *T*_c_. This is associated with the ability of this additive to nucleate in the PP matrix. In addition, as the wH content increased, the Δ*H*_m_ and Δ*H*_c_ levels tended to decrease because of the decreasing polymer amount.

The crystallinity (χ_c_) of PLA can be obtained using Equation 2.^47,48^
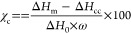
2where Δ*H*_m_ is the measured enthalpy of fusion and Δ*H*_0_ is the enthalpy of fusion for 100% crystalline PLA (Δ*H*_0_ = 93 J/g). Δ*H*_cc_ is the cold crystallization enthalpy, and ω is the weight fraction of PLA in the composite. The calculated χ_c_ values are shown in Table 5.

**Table 5:** Thermal Properties of wH/PLA Biocomposites

sample code	*T*_g_ (°C)	*T*_m_ (°C)	Δ*H*_m_ (J/g)	*T*_cc_ (°C)	Δ*H*_cc_ (J/g)	χ_c_
neat PLA	64.4	175.9	35.7	104.4	18.2	24.1
wH10-PLA	58.6	173.6	30.3	102.4	23.7	19.8
wH10-PLA-A	59.5	170.6	39.3	95.5	25.2	24.7
wH10-PLA-S	—	174.5	69.6	101.6	41.8	33.2
wH20-PLA	59.5	174.1	39.3	98.1	25.2	27.3
wH20-PLA-A	—	172.3	38.4	106.8	28.3	35.1
wH20-PLA-S	—	172.4	31.8	99.5	23.0	26.9
wH30-PLA	55.8	171.1	35.0	96.1	19.4	16.7
wH30-PLA-A	—	171.8	23.7	89.9	14.9	15.7
wH30-PLA-S	—	176.5	30.7	107.8	15.4	11.1
wH40-PLA	63.4	171.1	24.2	—	—	43.4
wH40-PLA-A	—	170.2	30.3	—	—	54.3
wH40-PLA-S	—	173.9	26.8	—	—	48.0

For the PLA biocomposites, similar to the PP biocomposites, the wH pretreatments did not have significant effects on the thermal properties (Table 5). A decrease in Δ*H*_m_ and Δ*H*_cc_ values was observed, especially at 30 and 40% wH contents. Moreover, with a decrease in the amount of PLA in the composite structure of the wH40 samples, the cold crystallization peak specific for PLA was lost. Therefore, an increase in the crystallinity was observed.

**Figure 6:**
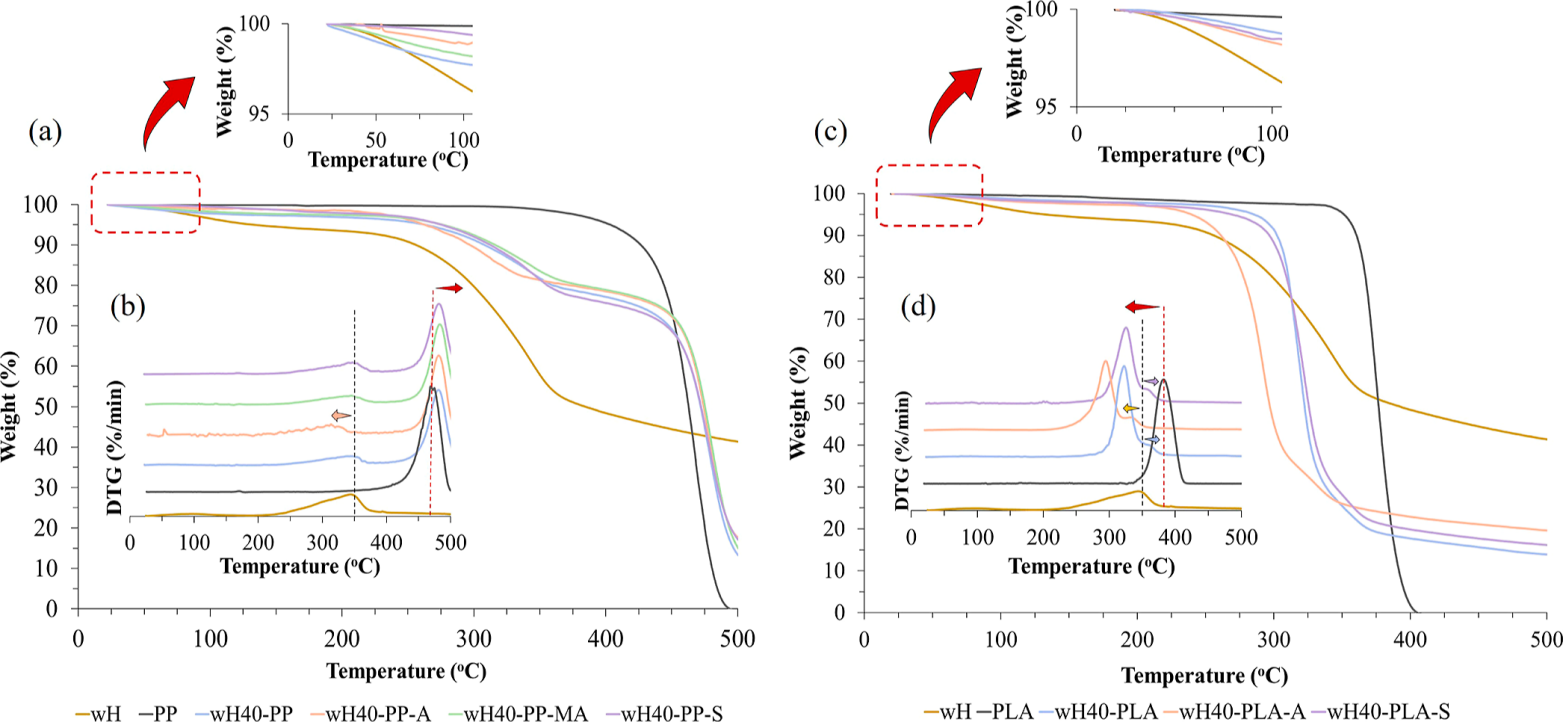
Thermograms: (a) TGA and (b) DTG curves for the wH40/PP biocomposites; (c) TGA and (d) DTG curves for the wH40/PLA biocomposites with different pretreatments.

As discussed in the next section, among the composites, the wH40/PP and wH40/PLA samples containing the highest wH content in each series showed the highest mechanical performance. For this reason, their thermal behaviors were investigated in detail at high temperatures by TGA. The TGA curves of the samples are shown in Figure 6a–d. Table 6 summarizes the main parameters obtained from the TGA curves, that is, the weight loss at temperatures of 10 and 50%, weight loss at 490 °C, and % moisture content. Figure 6a,b shows the thermal behavior of the wH40/PP samples. Two peaks were observed in the DTG curves of all biocomposite samples (Figure 6b). Compared to neat PP, there is a weight loss in the composite samples at 350 °C concerning cellulose decomposition in wH associated with the first peak.^49^ The second peak is also related to the decomposition of PP.^50^Figure 6a also depicts the difference in cellulose structure in the alkali-pretreated sample; cellulose degradation shifted to a lower temperature than the others. This is because only the alkaline pretreatment changed the structure of the wH surface. In this case, the thermal resistance of wH decreased. Moreover, the second peak of the samples shifted toward higher temperatures than that of PP. This indicates that the addition of the wH increased the thermal stability of PP in the composite.

**Table 6:** Main Thermal Parameters Obtained from TGA of the wH40/PP and wH40/PLA Composites with Different Pretreatments

sample code	*T*_weight loss_ (°C)		weight loss (%) at 490 °C	moisture (%)
	10%	50%		
wH	256	385	58	3.8
PP	427	463	99	0.1
wH40-PP	308	472	79	2.3
wH40-PP-A	297	474	76	1.0
wH40-PP-MA	316	475	76	1.8
wH40-PP-S	312	472	76	0.6
PLA	361	376	100	0.4
wH40-PLA	302	322	86	1.2
wH40-PLA-A	261	297	80	1.8
wH40-PLA-S	297	325	84	1.5

The PLA biocomposites showed a thermal behavior different from that of the PP biocomposites (Figure 6c,d). The first peaks of the biocomposites originated from PLA decomposition (Figure 6d).^51^ They shifted to a lower temperature than that of pure PLA. This shift indicates that the PLA in the composite underwent thermal degradation at a lower temperature than that of pure PLA. Similarly, the wH40-PLA-A sample showed cellulose decomposition at a lower temperature than the others owing to the effect of alkaline pretreatment on the wH structure.

The alkali-pretreated sample also has the lowest decomposition temperature at 10 and 50% weight losses (Table 6). The weight losses of all biocomposites were less than those of neat matrices with the effect of wH in the composite structure at 490 °C. The temperature at which 50% weight loss occurred was different for the PP and PLA biocomposites. For PLA biocomposites, this temperature is lower than that of pure PLA and wH, but for PP biocomposites, it is higher than that of pure PP and wH. In addition, Table 6 shows the % moisture content removed from the structure up to 105 °C, which was determined by TGA.

Although the % moisture content of the biocomposites was quite low, there was a decrease in the pretreated samples for the PP biocomposites. The silane-pretreated PP biocomposites exhibited the lowest moisture content compared to those of other samples, indicating improved moisture resistance. However, no significant difference in moisture content was observed among poly(lactic acid) (PLA) biocomposites.

### 4.3 Mechanical Test Results

**Figure 7:**
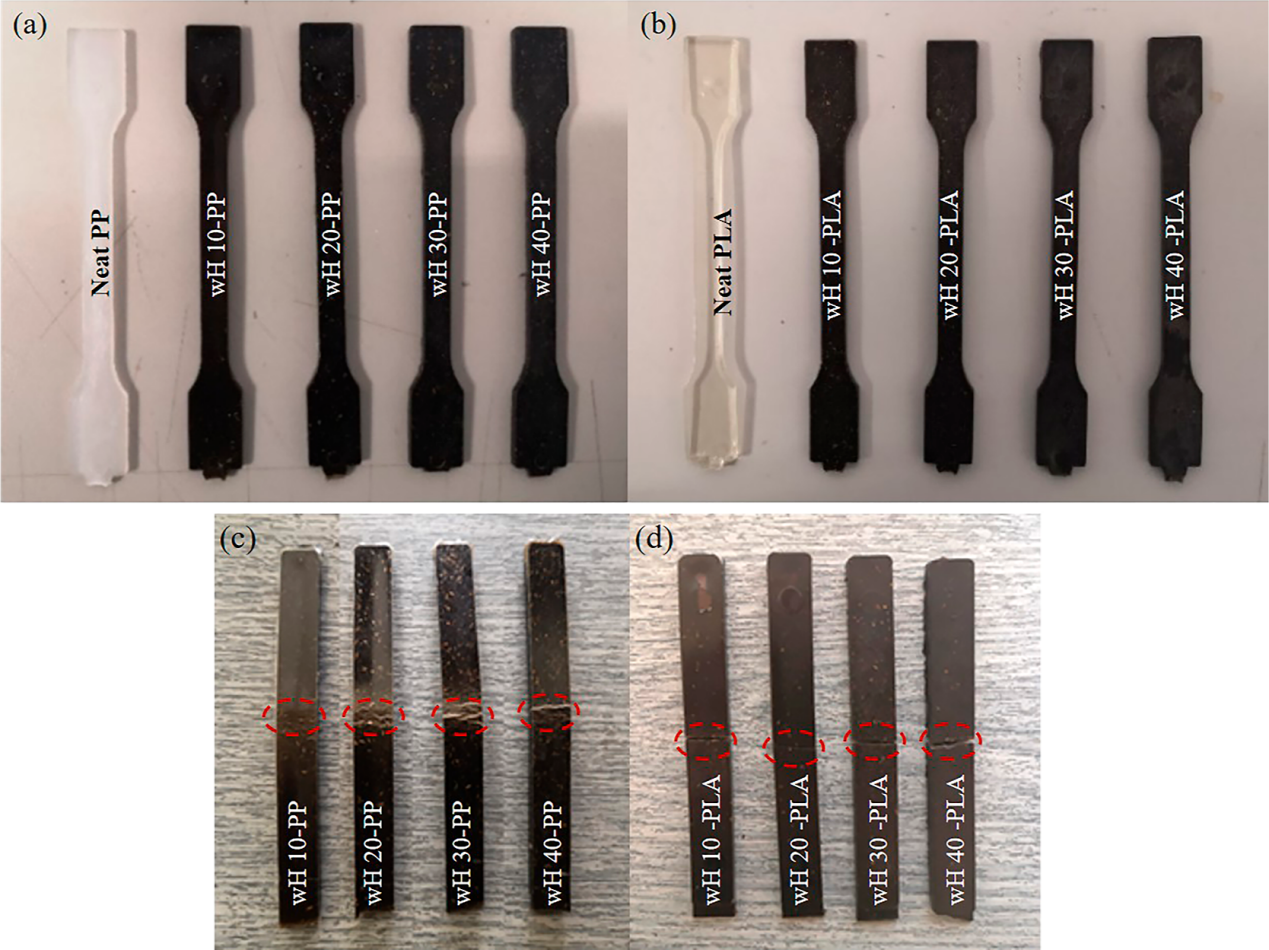
Tensile test specimens with an increasing amount of reinforcement (a) PP and (b) PLA samples; bending test specimens after test (c) PP and (d) PLA samples.

The mechanical performance of the reinforced biocomposites was determined through mechanical tests. The mechanical properties of the composites were investigated with respect to tensile strength, Young’s modulus, flexural strength, and flexural modulus. Figure 7a,b illustrates the injection-molded standard test specimens, where the reinforced samples exhibit a visually distinct woody appearance characterized by a darker color compared to the pure specimens. Notably, even with a mere 10% reinforcement, the material structure achieved a remarkably natural aesthetic without the incorporation of any color additive.

**Figure 8:**
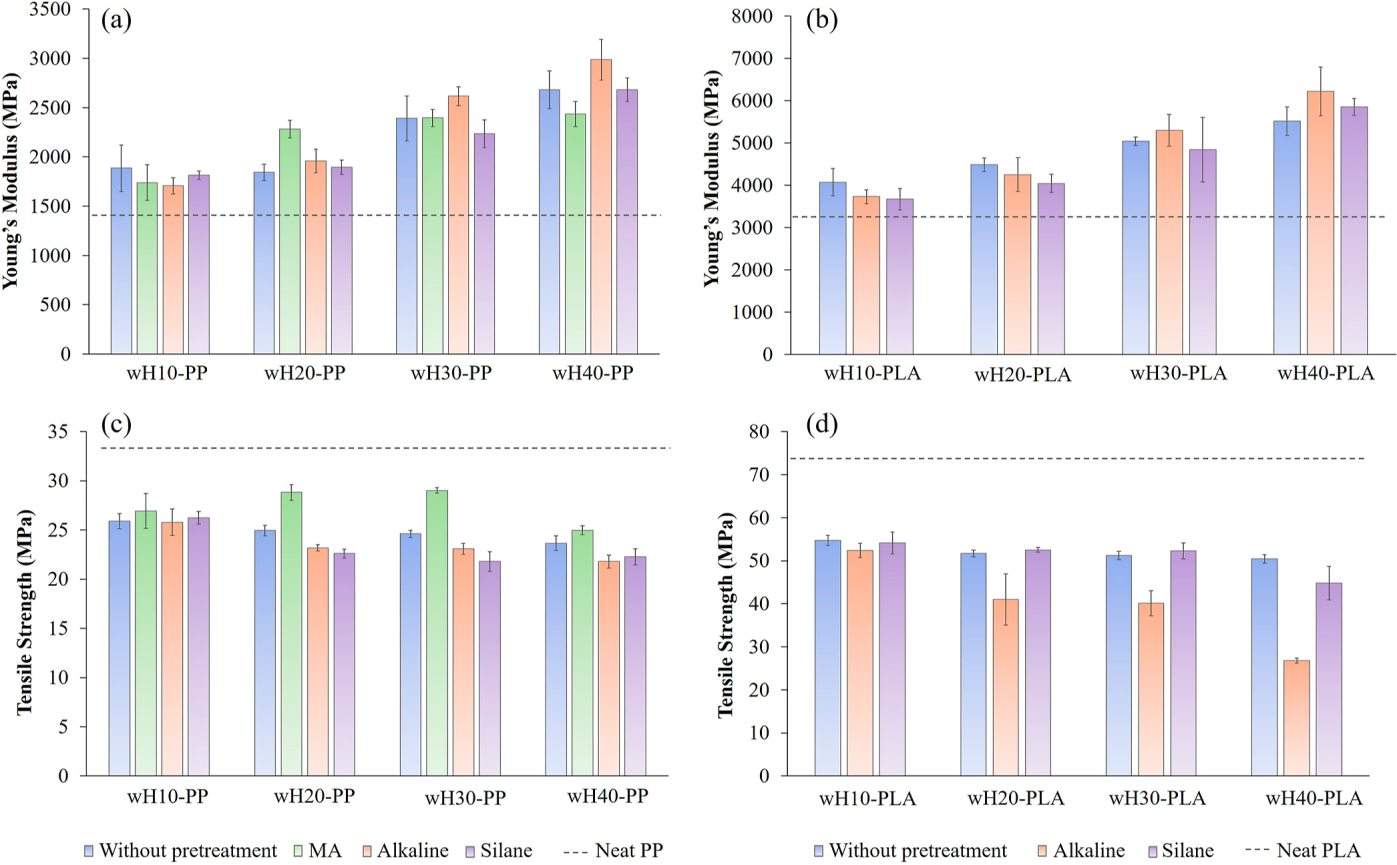
Tensile properties of biocomposites: Young’s modulus of (a) wH/PP and (b) wH/PLA; tensile strength of (c) wH/PP and (d) wH/PLA.

The tensile properties, including Young’s modulus and tensile strength, of all prepared biocomposites are shown in Figure 8. Similar to the results obtained by several researchers,^7,52^ in our study, Young’s modulus tended to increase with increasing wH content in both PP and PLA matrices. Pilla et al.^53^ examined the pine wood flour filler to the PLA matrix. As reported in their study, adding a natural filler to a polymeric structure restricts the movement of its chains, thereby increasing stiffness. These samples also had a higher tensile modulus than the neat matrices. Furthermore, the increase in stiffness was thought to be related to the effect of the compatibilizer on interfacial adhesion.^54^ The alkaline pretreatment led to the highest tensile modulus for the 30 and 40% wH contents. This is because the wH structure gains stiffness by removing hemicellulose and lignin in wH with alkaline pretreatment. This was supported by the data obtained from the FTIR analysis (Figure 3b), Figure 5b, and the van Soest method (Table 3). In addition, as these binding materials in the structure are removed, the fiber bundles turn into individual fibers, supported by the SEM image (Figure 5b). Apart from interface compatibility, this increased the module, which is similar to the findings of Mazzanti et al.^8^ The wH40-PP-A (2986 MPa) and wH40-PLA-A (6214 MPa) biocomposites exhibited the highest Young’s moduli, which were 109 and 81% higher than those of the pure polymers, respectively. The tensile strengths of both PP and PLA biocomposites were slightly lower than those of their pure polymers, as reported in previous studies.^54−56^ This may be due to the agglomeration of the wHs dispersed in the polymer matrix.^57^ Therefore, since the stress transfer in the biocomposite structure was blocked by the added wH, the local tension of the corresponding composite material increased and the structure became brittle; hence, the tensile strength decreased. In addition, because wH is used, its strength is lower than that of commercial hemp. Thus, the strength reduction, particularly in the pretreated biocomposite samples, can be attributed to this. It was observed that the MA-g-PP additive had a different effect on the tensile strength compared to other pretreatments in the PP biocomposites (Figure 8c). This is due to the long-chain polymeric additive that was introduced into the structure, which surrounded the wH and possibly reduced the agglomeration, thus making the overall biocomposite material less brittle. The wH exhibited increased rigidity and reduced flexibility as a consequence of alkaline pretreatment, which led to the removal of lignin and hemicellulose from its structure. Therefore, a decrease in tensile strength was most notable in alkaline-pretreated wHs, especially in wH/PLA samples with a high wH content. The PLA biocomposites showed the same trend as the PP biocomposites, but the tensile strengths of the wH-PLA-A samples, especially 40%, were significantly lower than those of the other samples.

**Figure 9:**
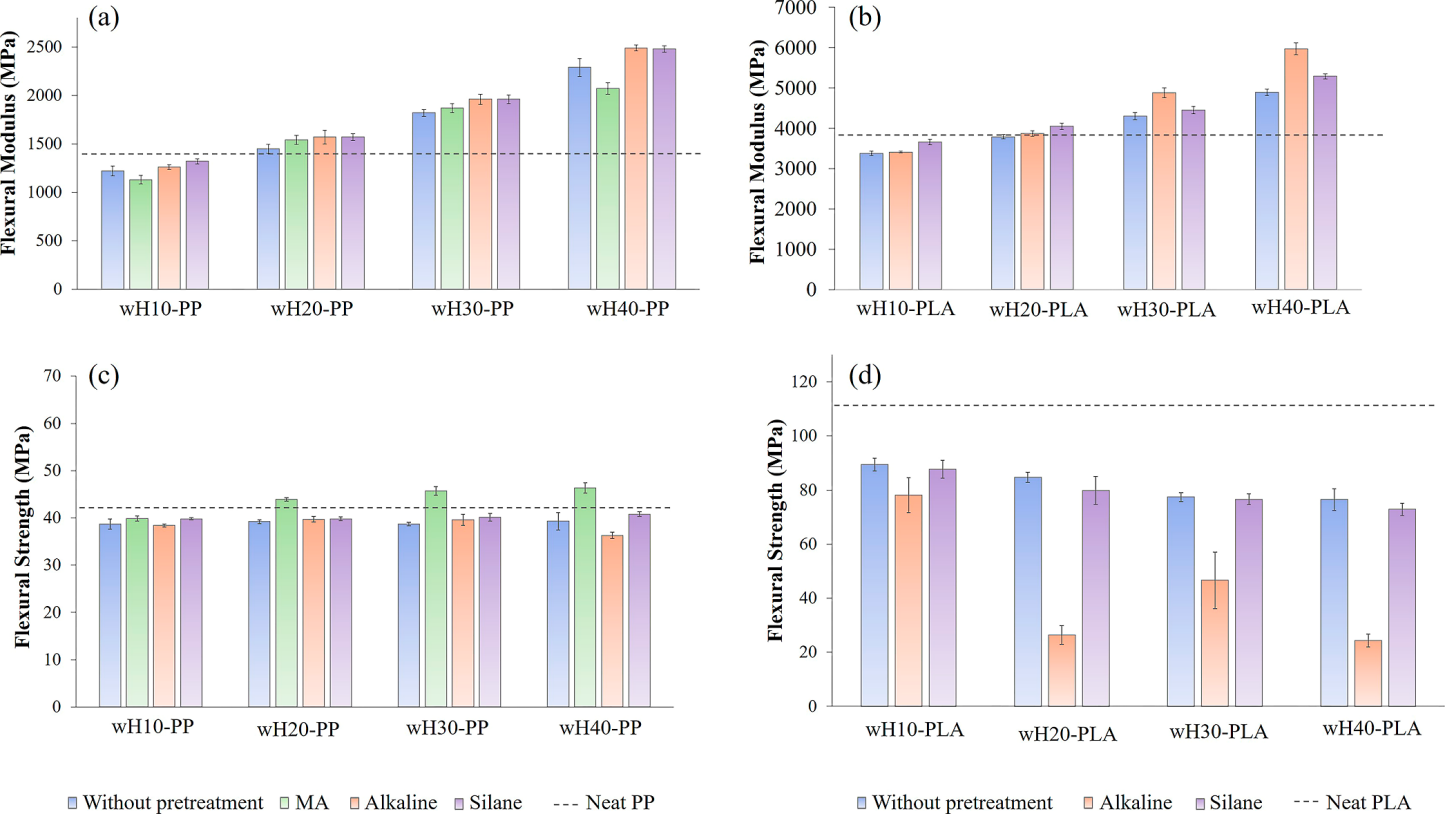
Bending properties of biocomposites: flexural modulus of (a) wH/PP and (b) wH/PLA; flexural strength of (c) wH/PP and (d) wH/PLA.

Figure 9 displays the bending properties of all of the prepared biocomposites, including the flexural modulus and flexural strength. The bending properties of the samples have the same tendency as the tensile properties and therefore can be interpreted similarly. Due to the high stiffness property of the wH, the flexural modulus increased similarly to the tensile modulus with increasing hemp content in the composite structure. It is considered that for this high stiffness to affect the overall composite structure, it has to be compatible with the wH and the matrix. Therefore, the effect of pretreatments can be observed particularly at high wH content. The flexural modules were found to be higher than 40% wH contents compared to the others. The high flexural modulus belonged to wH40-PP-A (2490 MPa) and wH40-PP-S (2480 MPa) for the PP biocomposites and wH40-PLA-A (5970 MPa) for the PLA biocomposites. These were 77, 76, and 56% higher than those of pure polymers, respectively. As the MA-g-PP additive decreased the stiffness of the material, the bending strength was found to be higher than those of the other pretreatments as well as the tensile strength. Similar to the tensile strength of the PLA biocomposites, alkaline pretreatment significantly reduced the flexural strength at wH contents of ≥20%.

Figure 7c,d shows that the bending behavior of the PP and PLA biocomposites changes with increasing wH content from 10 to 40%. The increase in the number of modules in Figure 9 is consistent with the structure of the samples obtained after testing. As the stiffness of the material increased, brittle fractures occurred.

## 5 Conclusions

This study successfully prepared wH-reinforced PP and PLA biocomposites via extrusion and injection molding processes. SEM images revealed that the pretreatments employed in this study enhanced the interfacial compatibility of the wH matrix. Regarding the thermal properties, only MA-g-PP increased *T*_c_. This difference was detected because the additive was directly added to the matrix and not to the wH, unlike in the other cases. In PP biocomposites, the addition of wH leads to an increase in the thermal resistance of the composite at high temperatures. However, the thermal resistance was unaffected by the addition of wH to the polymer matrix up to 220 °C. Mechanical properties were determined by using tensile and bending tests. Young’s modulus and flexural modulus increased with increasing wH content for both PP and PLA biocomposites. For PP biocomposites, the highest Young’s modulus (2986 MPa) and flexural modulus (2490 MPa) belonged to wH40-PP-A. These values were approximately 109 and 77% higher than those of neat PP, respectively. Similarly, wH40-PLA-A exhibited the highest number of modules among the PLA biocomposites tested. The Young’s modulus was 6214 MPa and the flexural modulus was 5970 MPa, which were about 81 and 56% higher, respectively, than those of the neat PLA. These findings provide valuable insights into the mechanical and thermal behaviors and compatibility of wH-reinforced PP and PLA biocomposites, promising for many applications.

## Supporting Information Available

Proximate analysis of the wH and optical microscope image of a lignocellulosic fiber in the wH (PDF)
