# Supplementary material for: Utilizing the Potential of Waste Hemp Reinforcement: Investigating Mechanical and Thermal Properties of Polypropylene and Polylactic Acid Biocomposites
Source: ACS Omega. 2024 Feb 14;9(8):8818–28. doi: 10.1021/acsomega.3c06240 (PMC10905589; doi:10.1021/acsomega.3c06240)
Supplement: Supplementary file 1 — ao3c06240_si_001.pdf [file ao3c06240_si_001.pdf]

Supporting Information

Utilizing the Potential of Waste Hemp Reinforcement: Investigating Mechanical and Thermal Properties of Polypropylene and Polylactic Acid Biocomposites

Anıl Yılmaz,*[a] Hakan Özkan,[b] and F. Elif Genceli Güner,[ac]

a. Department of Chemical Engineering, Istanbul Technical University, Maslak 34469 Istanbul, Turkey
b. Arçelik Çayırova Campus, R&D Material Technologies, R&D Center, 34950, Istanbul, Turkey
c. Polar Research Center (PolReC), Istanbul Technical University, 34469, Maslak, Istanbul, Turkey
* Corresponding author

Table of Contents

Table S1. Proximate analysis of the wH.

Figure S1. Optical microscope image of a lignocellulosic fiber in the wH.

Table S1. Proximate analysis of the wH.

Proximate analysis	wt%
Moisture	8.6
Volatiles	71.9
Fixed carbon	4.7
Ash	14.7

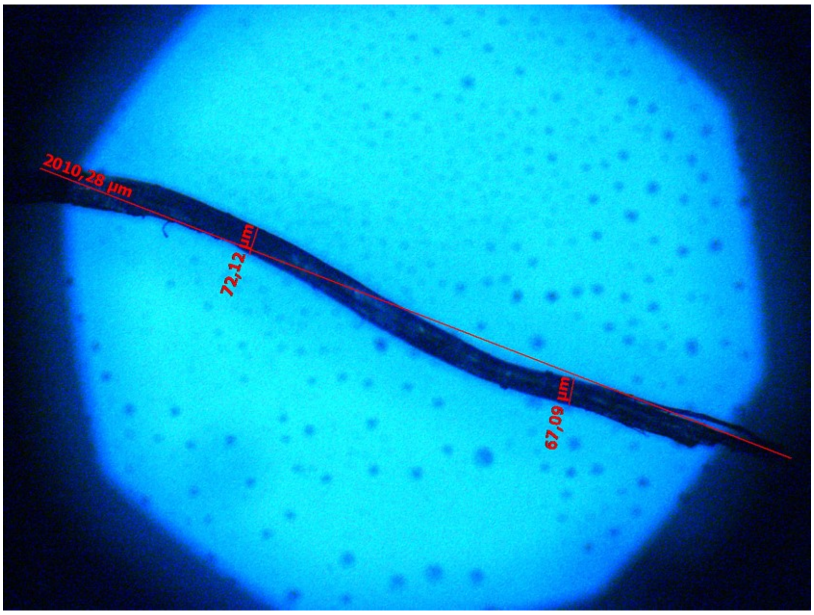

Figure S1. Optical microscope image of a lignocellulosic fiber in the wH.
